# Molnupiravir Real-World Utilization in COVID-19 Patients in the Czech Republic

**DOI:** 10.3390/jcm13082303

**Published:** 2024-04-16

**Authors:** Pavel Dlouhý, Cyril Mucha, Lenka Mokrá, Matyáš Kuhn, Lenka Hrdlickova, Urs Arnet, Yohance Whiteside

**Affiliations:** 1 Infectious Disease Department, Krajská Zdravotní, a.s., 400 11 Ústí nad Labem, Czech Republic; 2 Institute of General Practice, First Faculty of Medicine, Charles University, 110 00 Prague, Czech Republic; 3 Institute of Biostatistics and Analyses, Ltd., 625 00 Brno, Czech Republic; 4 MSD Czech Republic, 150 00 Prague, Czech Republic; 5 Center for Observational and Real-World Evidence (CORE), MSD Innovation & Development GmbH, 8058 Zürich, Switzerland; urs.arnet@msd.com; 6 Center for Observational and Real-World Evidence (CORE), Merck & Co., Inc., Rahway, NJ 07065, USA

**Keywords:** molnupiravir, real-world data, COVID-19, Czech Republic, treatment of COVID-19, SARS-CoV-2

## Abstract

**Background/Objectives**: Molnupiravir (MOV), an oral antiviral COVID-19 treatment, was introduced in the Czech Republic in December 2021 for COVID-19 patients at a high risk of progression to severe disease requiring hospitalization. In this observational, retrospective study, we aimed to describe the characteristics and healthcare resource utilization in non-hospitalized, adult COVID-19 patients prescribed MOV in the Czech Republic between 1 January and 30 April 2022. **Methods**: A total of 621 patients were included and followed up with for 28 days. **Results**: The median age was 68.0 (20–99) years, 77.8% were overweight or obese, 14.1% smoked, and 85.7% were vaccinated. The overall cumulative incidence (95% CI) of all-cause hospitalization was 0.71 (0.37; 1.24) per 1000 person years or 1.9%, with similar rates across sexes, age groups, BMI category, multimorbidity category, polypharmacy category, and COVID-19 vaccination status. Among patients reported hospitalized, oxygen-based resources were not observed, and no deaths occurred. **Conclusions**: These data describe the characteristics and healthcare resource utilization in Czech MOV-treated patients whose clinical characteristics may put them at increased risk of severe disease.

## 1. Introduction

In late 2019–early 2020, a respiratory disease caused by a novel coronavirus, SARS-CoV-2 (COVID-19), started spreading rapidly across the globe and resulted in a pandemic [1,2]. Although the public health emergency ended on 5 May 2023, new cases are still being reported and long-term management measures are recommended [3]. The COVID-19 disease ranges from asymptomatic or mild to severe respiratory syndrome and may be lethal. By 7 May 2023, 765,903,278 cases including 6,927,378 deaths have been reported to the WHO [4]. The COVID-19 pandemic overwhelmed healthcare systems around the world, and the admission of patients to general wards and intensive care units (ICUs) caused a significant drain on healthcare resources [5]. In addition, COVID-19 significantly impacted the global economy, resulting in substantial direct costs for diagnosing and treating patients and indirect costs related to loss of income and productivity [6,7]. While the impact of the first pandemic wave was low in the Czech Republic due to the early implementation of very restrictive measures [8], during the second wave, the mortality was highest in central and eastern Europe, including Poland, Bulgaria, Slovenia, Czechia, Romania, and Hungary [9]. 

Vaccines helped to reduce its associated morbidity and mortality [10]. However, in the first half of 2022, according to the European Centre for Disease Prevention and Control (ECDC), the vaccination rate in the Czech Republic was lower compared to other European Union Member States, namely, 64.5% of the total population had received two doses and 40.8% had received a booster compared to the EU average of 73.1% and 54.8%, respectively [11]. Thus, the availability of effective therapies to treat COVID-19 was in the Czech public health interest.

Molnupiravir (MOV) is an orally bioavailable ribonucleoside analog with broad-spectrum activity against COVID-19 [12]. In a MOVe-OUT clinical trial, MOV was shown to significantly reduce the risk of COVID-19 hospitalizations or mortality in high-risk patients when administered within 5 days of symptom onset [13]. Individuals treated with MOV had a significant reduction in the risk of hospitalizations and/or deaths from 9.7% in the placebo group to 6.8% in the MOV group [13]. In December 2021, the drug was temporarily authorized by the Czech Ministry of Health (based on §11 of Act No. 378/2007) to be prescribed for the treatment of mild to moderate COVID-19 disease in adults with a positive SARS-CoV-2 diagnostic test who were at a high risk of progression to severe disease necessitating hospitalization [14].

In this observational study, we aimed to describe the characteristics, real-world clinical outcomes, and healthcare resource utilization (HCRU) in Czech patients who were prescribed MOV between 1 January and 30 April 2022, and were followed up with for 28 days. During this study period, MOV was the only antiviral available for treatment for SARS-CoV-2 in the Czech Republic. Since MOV was previously shown to significantly reduce the risk of COVID-19 hospitalizations and mortality for high-risk patients [13], and MOV was the only antiviral drug available for COVID-19 treatment in the Czech Republic, this study did not seek to assess comparative effectiveness but sought to describe real-world outcomes.

## 2. Materials and Methods

### 2.1. Data Source

This was a retrospective study using structured data. In the Czech Republic, no structured COVID-19 patient registries were available for real-world evidence (RWE) generation. To collect these data, general practitioners entered anonymized (de-identified) patient-level data from their medical records (electronic and/or paper charts) into CLADE-IS, an electronic data capture (EDC) and electronic case report form (eCRF) system developed by the Institute of Biostatistics and Analyses (IBA). Patient charts in the healthcare provider (HCP) office included all relevant data such as medical history, patients’ visits, hospital referrals, insurance correspondence, etc.

### 2.2. Study Population

The study population included non-hospitalized, adult (age ≥ 18 years) COVID-19 patients who were prescribed MOV between 1 January 2022 and 30 April 2022, and who had a confirmed positive SARS-CoV-2 polymerase chain reaction (PCR) test or a positive COVID-19 antigen test between 27 December 2021 and 30 April 2022 recorded in a medical facility within 5 days before the first MOV prescription. Patients had to be non-hospitalized (defined as any visit that was not an inpatient hospitalization) on the index date. Individuals with evidence of pregnancy on or within 3 months before the index date or with evidence of hospitalization on the index date were excluded.

### 2.3. Variables

#### 2.3.1. Patient and Clinical Characteristics

The primary objective was to describe the patient and clinical characteristics of non-hospitalized, adult COVID-19 patients prescribed MOV in a real-world clinical practice. To analyze the primary objective, we collected and analyzed data of our target patient population. We defined the index date as the first MOV prescription date recorded in the patient chart; this was considered the treatment start date. The baseline period spanned from 1 day to 12 months before the index date and was used to describe patient and clinical characteristics. Patients’ characteristics included age and sex. Clinical characteristics included body mass index (BMI), comorbidities, concomitant medications, COVID-19 vaccination status, vaccination type, diagnostic test type, and variant status. BMI was classified as underweight, healthy weight, overweight, and obese, using BMI kg/m^2^ cutoffs of <18.5, 18.5–24.9, 25–29.9, and ≥30.0, respectively. Comorbidities were identified using the International Classification of Diseases 10th Revision (ICD-10) and were collected during 12 months before the index date (Supplementary Table S1).

Comorbid conditions included those associated with an increased risk of severe COVID-19 disease according to the Centers for Disease Control (CDC) and Prevention 2021 list and additional risk factors designated by the Czech Republic Ministry of Health [14,15]. Multimorbidity was defined as the presence of two or more comorbidities. Concomitant medication data were collected within 90 days before the index date and included medications that had potential contraindicated or major drug–drug interactions with ritonavir-containing COVID-19 therapy. (Supplementary Table S2) [16,17,18]. COVID-19-related characteristics at the index date included vaccination status (vaccinated, defined as having received at least one dose of any COVID-19 vaccine, versus non-vaccinated), vaccination type (Comirnaty (Pfizer—BioNTech, Pfizer HQ, New York, NY, USA), Spikevax (Moderna—NIAID, Bethesda, MD, USA), Vaxzevria (AstraZeneca—Oxford, UK), Jcovden (Janssen Pharmaceuticals, Titusville, NJ, USA), Nuvaxovid (Novavax, Gaithersburg, MD, USA), other, unknown), test type including date of test (PCR test, antigen test), and SARS-CoV-2 variant.

#### 2.3.2. Healthcare Resource Utilization (HCRU)

Exploratory objectives were included to assess the health outcomes and HCRU in our patient population. HCRU outcomes, measured during the 28-day follow-up period after the index date, included all-cause hospitalization and all-cause mortality. All-cause hospitalization was defined as any inpatient visit that was longer than 24 h. All-cause mortality was obtained from eCRF entries based on the records in medical charts. Outcomes included all-cause hospitalization and all-cause mortality due to concerns about the completeness of data on COVID-19-related hospitalizations and deaths. To account for potential data lag, we included a four-week extension after the follow-up period to collect data and assess the outcomes. 

HCRU during the 28-day follow-up period was described in terms of the frequency of outpatient and inpatient visits. Outpatient visits included emergency room visits (ER), physician office or hospital outpatient visits, telehealth/telephone visits, or email/mail communication. They also included data about post-index SARS-CoV-2 tests, test results, and the number of RT-PCR, antigen and/or antibody tests. Inpatient visits were described as the number of hospital and ICU stays and the cumulative length of stay (LOS), and included data about oxygen administration, invasive mechanical ventilation (IMV), and/or extracorporeal membrane oxygen (ECMO), and number of intervention administration days.

### 2.4. Statistical Analysis

Descriptive statistical results were reported. Numbers and percentages were provided for dichotomous and categorical variables. Continuous variables were summarized as median and inter-quartile range (IQR), with minimum and maximum values. Since this was a descriptive study, no formal power calculations were made. Cumulative incidence rates were reported with a 95% confidence interval (CI). All analyses were performed using R version 3.6.3.

## 3. Results

### 3.1. Construction of Study Population

A total of 621 patients received a prescription for MOV, met the inclusion criteria, and were followed up with for 28 days after the index date. In 29 patients (4.7%), the study outcomes were assessed prior to the 28-day follow-up period completion. However, a sensitivity analysis showed that there were no significant differences in the outcomes when these individuals were included in the analysis. On average, these patients were slightly younger and more likely non-vaccinated. These differences were not statistically significant and had no impact on the analysis.

### 3.2. Baseline Demographics

Baseline demographics of patients in the study are described in Table 1. In the 621 participants, the median age was 68.0 (20–99) years, and 54.3% were females. At the index date, 77.8% were overweight or obese, and 14.1% of patients smoked. 

In most patients (88.9%), less than 3 days elapsed between receiving a positive SARS-CoV-2 diagnostic test and receiving a prescription for MOV. Most patients were vaccinated. The most common comorbidities included hypertension (71.0%), obesity (43.3%), and diabetes mellitus (25.6%). Multimorbidity was present in 71.3% of patients. The most common concomitant medications included antihypertensives (69.9%), HMG-CoA reductase inhibitors (statins) (39.6%), and antiglycemics (22.1%). Polypharmacy was observed in 25.1% of patients. Additional demographic data, comorbidities, and concomitant medications among patients can be found in Supplementary Tables S1, S2 and S3, respectively.

### 3.3. Hospitalization and Mortality

Cumulative incidence rates for all-cause hospitalization and all-cause mortality during the 28-day follow-up period are summarized overall and by risk factors in Table 2. The overall cumulative incidence rate of all-cause hospitalization (95% CI) during the follow-up period was 0.71 (0.37, 1.24) or 1.9%, with similar rates across age groups, sexes, BMI category, multimorbidity category, pharmacy category, and vaccination status. None of the patients treated with MOV died during the follow-up period, resulting in a cumulative incidence rate of 0% for all-cause mortality.

### 3.4. Healthcare Resource Utilization

Out of the 621 patients who received MOV, 1.1% of patients visited an ER during the follow-up period, resulting in a rate of 0.41 per 1000 person days (Table 3). Less than 10% of patients required an outpatient hospital/physician office visit or a telehealth or telephone visit. Email/mail communication occurred with less than 1% of patients. Two (0.3%) patients required intensive care hospitalization; the rate was 0.12 (0.01; 0.43) per 1000 person days. The median length of stay in days was 5.5 (IQR = 3.0–16.5). Less than 1% of patients had a post-index SARS-CoV-2 test. Less than 1% of patients were administered oxygen. For those requiring supplementary oxygen administration, the length of administration was recorded for one patient and lasted 13 days. No patients required IMV or ECMO.

All-cause hospitalizations were observed in 12 patients (see Table 2).

## 4. Discussion

This study describes the characteristics, healthcare resource utilization, and outcomes for patients treated with MOV during the COVID-19 pandemic in the Czech Republic in early 2022. Forty-five percent (45.6%) were ≥70 years of age, 54.3% were females, 71.3% had ≥2 comorbidities that put them at increased risk of severe COVID-19, and 73.1% were taking ≥1 comedication that was either contraindicated to or had major potential for drug–drug interactions with ritonavir-containing COVID-19 therapy. In this study, the proportion of all-cause hospitalizations was 1.9%, and no deaths were recorded.

Because of the effectiveness of MOV in reducing the risk of COVID-19 hospitalizations and mortality has been established [13], this study focused on describing the characteristics, real-world clinical outcomes, and healthcare resource utilization (HCRU) of users. It was designed as a single-arm study because MOV was the only antiviral drug available for COVID-19 treatment in the Czech Republic during the study period. Further complicating any attempt at comparative effectiveness is the positioning between nirmatrelvir/ritonavir and MOV in COVID-19 treatment guidelines. Channeling bias may result where MOV is generally prescribed to more medically complex patients who are unable to be treated with nirmatrelvir/ritonavir, placing them at higher risk of severe COVID-19 disease. 

Prior to these findings, other single-arm real-world studies had described the utilization of MOV. De Vito et al. [19] described the safety and efficacy of MOV in a population with a high comorbidity burden that was slightly older (mean age 70.4 year vs. 66.2 years) and more likely male (54.5% vs. 45.4%) than ours. A study conducted by Streinu-Cercel [20] in Romania reported improvement in symptoms and no worsening in severity among a MOV-treated population that was much younger than those included in this analysis (median age 45.8 years vs. 68.0 years) but had a similarly high rate of COVID-19 vaccinations. Kimata et al. [21] demonstrated the safety and effectiveness of MOV in a similar population of older patients (median age 68.0 years) but with a lower median BMI (23.5 vs. 28.7) than our Czech patient population. Prajapati et al. [22] described MOV users that were younger than those included in this study (median age 59.0 years vs. 68.0 years) with fewer having ≥2 comorbidities that placed them at increased risk of severe COVID-19 (57.8% vs. 71.3%). 

During the study period, the number of individuals diagnosed with COVID-19 in the Czech Republic was 1,336,646, the hospitalization rate was 2.63% (35,217), and the COVID-19 mortality rate was 0.3% (3940) in individuals ≥18 years old [23]. In the age group ≥ 65 years old, the number of positive cases was 147,800, the number of hospitalizations was 24,966 (16.9%), and the number of deaths was 3489 (2.36%) [22]. Although our study population is not a representative sample of the population aged 65 years and older, the 1.9% observed cumulative incidence rate for all-cause hospitalization in MOV-treated patients was less than the COVID-19 hospitalization rate (2.63%) during the same period. Even though the overall hospitalization rate in the Czech Republic cannot be compared with our study sample [23], it allows us to conclude that the hospitalization rate for MOV-treated COVID-19 patients treated in this setting was low. The observed hospitalization rates in our study were higher than in the PANORAMIC study [24]. However, the COVID-19 severity and outcome differed during the same period in the Czech Republic compared to the UK pandemic situation [24]. The patient population receiving MOV in PANORAMIC were not consistent with the population in the Czech Republic. 

Several limitations should be noted. As in any retrospective observational study, we cannot exclude an inherent bias due to potentially missing data. Since this was a single-arm study, we could not evaluate the effectiveness of MOV. Since the number of patients in this study was low, the results may not be generalizable to all Czech COVID-19 patients who received MOV. This analysis may have missed hospitalizations outside of patient charts and those not reported to physicians. Hospitalization was based on all-cause hospitalization and could not reliably be attributed to COVID-19-related hospitalization. Outcomes included all-cause hospitalization and all-cause mortality due to concerns about the completeness of data on COVID-19-related hospitalizations and deaths. In addition, the data used in this analysis could not be used to confirm with certainty that MOV administration started within 5 days of symptom onset. Finally, a prescription for MOV does not ensure its use.

## 5. Conclusions

This study seeks to shed light on the characteristics, real-world clinical outcomes, and healthcare resource utilization for individuals who are prescribed MOV for COVID-19. These data are important because they provide additional context to the growing body of evidence on the utility of MOV, by providing a valuable country-level perspective on a population with a high rate of comorbidities. In our population of older patients with increased risk of severe disease, we observed a 0.71% all-cause hospitalization rate and a 0% all-cause mortality rate. While the absence of a comparison group precludes conclusions regarding treatment effectiveness, these data describe the characteristics and outcomes in Czech COVID-19 patients treated with MOV. 

## Figures and Tables

**Table 1:** Characteristics of patients treated with MOV in the Czech Republic, 1 January 2022–30 April 2022 (*N* = 621).

Patient Characteristics	*N* = 621
**Age (years)**	
Median (Min; Max)	68.0 (20; 99)
IQR	58.0; 76.0
Mean (SD)	66.2 (14.39)
**Age group**	
18–49	82 (13.2%)
50–59	91 (14.7%)
60–69	165 (26.6%)
≥70	283 (45.6%)
**Sex**	
Male	282 (45.4%)
Female	337 (54.3%)
Unknown	2 (0.3%)
**BMI category**	
Underweight	6 (1.0%)
Healthy weight	132 (21.3%)
Overweight	214 (34.5%)
Obese	269 (43.3%)
**Smoking**	
Yes, more than 10/day	88 (14.1%)
No	447 (72.0%)
Unknown	86 (13.8%)
**Time between positive SARS-CoV-2 test and MOV start**	
0–2 days	552 (88.9%)
3–5 days	69 (11.1%)
**Vaccination status**	
No	89 (14.3%)
Yes	532 (85.7%)
**Three most common comorbidities**	
Hypertension	441 (71.0%)
Obesity	269 (43.3%)
Diabetes mellitus, type 1 and type 2	159 (25.6%)
**Multimorbidity**	
0–1 comorbid condition	178 (28.7%)
≥2 comorbid conditions	443 (71.3%)
**Three most common concomitant medications**	
Antihypertensives	434 (69.9%)
HMG-CoA reductase inhibitors (statins)	246 (39.6%)
Antiglycemics	137 (22.1%)
**Polypharmacy (≥5 comedications)**	
Yes	156 (25.1%)
No	465 (74.9%)

**Table 2:** Outcomes following molnupiravir (MOV) treatment in the Czech Republic, 1 January 2022–30 April 2022, overall and by risk factors.

	Total *N*	Cumulative Incidence (95% CI, 28 Days Post Index) per 1000 Person Days	
**Patient characteristic**		**All-cause hospitalization**	**All-cause mortality**
Overall	621	0.71 (0.37; 1.24) ^1^	0.00 (0.00; 0.22)
**Age group, years**			
18–49	82	0.91 (0.11; 3.29)	0.00 (0.00; 1.68)
50–59	91	0.40 (0.01; 2.24)	0.00 (0.00; 1.49)
60–69	165	0.89 (0.24; 2.27)	0.00 (0.00; 0.82)
≥70	283	0.65 (0.21; 1.51)	0.00 (0.00; 0.48)
**Sex**			
Male	282	0.52 (0.14; 1.33)	0.00 (0.00; 0.48)
Female	337	0.87 (0.38; 1.72)	0.00 (0.00; 0.40)
Unknown	2	0.00 (0.00; 65.87)	0.00 (0.00; 65.87)
**BMI category**			
Underweight	6	0.00 (0.00; 21.96)	0.00 (0.00; 21.96)
Healthy weight	132	0.57 (0.07; 2.06)	0.00 (0.00; 1.05)
Overweight	214	0.68 (0.19; 1.75)	0.00 (0.00; 0.63)
Obese	269	0.81 (0.30; 1.77)	0.00 (0.00; 0.50)
**Multimorbidity category**			
0–1	178	0.63 (0.13; 1.84)	0.00 (0.00; 0.78)
≥2	443	0.74 (0.34; 1.40)	0.00 (0.00; 0.30)
**Polypharmacy category**			
0–4	465	0.71 (0.33; 1.36)	0.00 (0.00; 0.29)
≥5	156	0.69 (0.14; 2.03)	0.00 (0.00; 0.85)
**Vaccination Status**			
No	89	0.84 (0.10; 3.05)	0.00 (0.00; 1.56)
Yes	532	0.69 (0.33; 1.26)	0.00 (0.00; 0.25)

^1^ 0.71 (0.37; 1.24) is the rate for any hospitalization (if patient had more than one hospitalization, both were included in the rate) during the whole follow-up period.

**Table 3:** Outpatient and inpatient healthcare resource utilization (HCRU) following molnupiravir (MOV) treatment in the Czech Republic, 1 January 2022–30 April 2022 (*N* = 621).

Healthcare Resource Utilization	Number of Patients with Visit *n* (%)	Cumulative Incidence (95% CI, 28 Days Post Index)
**Outpatient Resource Use**	**Data**	**Data**
Emergency room (ER) visit	7 (1.1%)	0.41 (0.17; 0.85)
Outpatient visit to a hospital/physician office	45 (7.2%)	4.90 (3.91; 6.08)
Telehealth or telephone visit	50 (8.1%)	5.38 (4.33; 6.60)
Email/mail communication	4 (0.6%)	0.53 (0.24; 1.01)
Additional SARS-CoV-2 tests during follow-up		
RT-PCR	4 (0.6%)	
*RT-PCR positive*	3 (75.0%)	
Antigen test	1 (0.2%)	
*Antigen test positive*	0	
Antibody test	1 (0.2%)	
*Antibody test positive*	1 (100.0%)	
**Inpatient resource use**		
Intensive care unit visit	2 ^1^	
Oxygen administration	2	0.12 (0.01; 0.43)
Invasive mechanical ventilation (IMV)	0	0.00 (0.00; 0.22)
Extra corporeal membrane oxygenation (ECMO)	0	0.00 (0.00; 0.22)

^1^ ICU stay was observed in two patients: 0.12 (0.01; 0.43)

## Data Availability

All data generated or analyzed during this study are included in this published article and Supplementary Materials.

## Supplementary Materials

The following supporting information can be downloaded at https://www.mdpi.com/article/10.3390/jcm13082303/s1: Table S1: Additional characteristics of patients treated with MOV in the Czech Republic, 1 January 2022–30 April 2022 (*N* = 621); Table S2: Additional comorbidities among patients treated with MOV in the Czech Republic, 1 January 2022–30 April 2022 (*N* = 621); Table S3: Additional concomitant medications with a high potential for drug–drug interactions with nirmatrelvir/ritonavir among patients treated with MOV in the Czech Republic, 1 January 2022–30 April 2022 (*N* = 621).
