# Supplementary material for: Molnupiravir Real-World Utilization in COVID-19 Patients in the Czech Republic
Source: J Clin Med. 2024 Apr 16;13(8):2303. doi: 10.3390/jcm13082303 (PMC11051211; doi:10.3390/jcm13082303)
Supplement: Supplementary file 1 [file jcm-13-02303-s001.zip › jcm-2905058-supplementary.pdf]

Table S1. Additional characteristics of patients treated with MOV in the Czech Republic, January 1, 2022 – April 30, 2022 (N=621)

**Patient characteristics**	**N=621**
**BMI (kg/m²)**	
N used	621
Median (Min; Max)	28.70 (15.9; 65.8)
IQR	25.40; 33.60
Mean (SD)	30.18 (7.086)
**Type of test used for COVID-19 diagnosis**	
RT-PCR	489 (78.7%)
Antigen test	132 (21.3%)
**COVID-19 variant**	
Omicron (All BA. 1 and 2)	84 (13.5%)
Delta	5 (0.8%)
Unknown	532 (85.7%)
**Time from last vaccination to baseline positive test (months)**	
N used	500
Median (Min; Max)	3.68 (0.2; 12.7)
IQR	2.53; 4.83
Mean (SD)	4.05 (2.268)
Missing	32
**Type of vaccination**	
Comirnaty (Pfizer/BioNTech),	361 (67.9%)
Spikevax (Moderna),	69 (13.0%)
Vaxzevria (AstraZeneca),	21 (3.9%)
Jcovden (Janssen Pharmaceutica),	9 (1.7%)
Other or mixed vaccine application	52 (9.8%)
Unknown	20 (3.8%)
**Type and number of vaccinations**	
Comirnaty (Pfizer/BioNTech)	N = 361
1	5 (1.4%)
2	68 (18.8%)
3	288 (79.8%)
Spikevax (Moderna),	N = 69
1	1 (1.4%)
2	11 (15.9%)
3	57 (82.6%)
Vaxzevria (AstraZeneca),	N = 21
1	1 (4.8%)
2	20 (95.2%)
Jcovden (Janssen Pharmaceutica)	N = 9

1	9 (100.0%)

Table S2. Prevalence of additional comorbidities among patients treated with MOV in the Czech Republic, January 1, 2022 – April 30, 2022 (N=621)

**Comorbidities**	**N=621**
Heart conditions	92 (14.8%)
Cancer	91 (14.7%)
Asthma	78 (12.6%)
Chronic lung diseases	68 (11.0%)
Chronic liver disease	60 (9.7%)
Neurological conditions including dementia	59 (9.5%)
Chronic kidney disease	57 (9.2%)
Mental health disorders	42 (6.8%)
Cerebrovascular disease	28 (4.5%)
Primary thrombophilia	24 (3.9%)
Substance use	16 (2.6%)
Obstructive and central sleep apnea	14 (2.3%)
Immune deficiencies	8 (1.3%)
Solid organ or blood stem cell transplantation	6 (1.0%)
History of recurrent thromboembolic events	2 (0.3%)
Sickle cell disease	0
Beta Thalassemia	0
Tuberculosis	0
HIV	0
**Number of comorbidities**	
0	36 (5.8%)
1	142 (22.9%)
2	184 (29.6%)
3	125 (20.1%)
4	71 (11.4%)
≥ 5	63 (10.1%)

Table S3. Additional concomitant medications with a contraindication to or potential for major drug-drug interactions with nirmatrelvir/ritonavir among patients treated with MOV in the Czech Republic, January 1, 2022 – April 30, 2022 (N=621)

**Concomitant medications**	**N=621**
Neuropsychiatric drugs	93 (15.0%)
Inhaled and oral corticosteroids	67 (10.8%)
Oral anticoagulants	61 (9.8%)
Corticosteroids and other immunosuppressive meds	42 (6.8%)
Antibiotics	34 (5.5%)

Antiarrhythmic agents	32 (5.2%)
Antineoplastics	19 (3.1%)
Immunosuppressants	17 (2.7%)
Anticonvulsants	9 (1.4%)
**Number of different concomitant medications**	
0	83 (13.4%)
1	84 (13.5%)
2	103 (16.6%)
3	118 (19.0%)
4	77 (12.4%)
≥ 5	156 (25.1%)
